# Supplementary material for: Ceramide Synthase 6 Maximizes p53 Function to Prevent Progeny Formation from Polyploid Giant Cancer Cells
Source: Cancers (Basel). 2021 May 5;13(9):2212. doi: 10.3390/cancers13092212 (PMC8125704; doi:10.3390/cancers13092212)
Supplement: Supplementary file 1 [file cancers-13-02212-s001.zip › cancers-1170757-supplementary.pdf]

Supplementary Figure S1. Metabolic analysis of parental and PGCC from PPC1 cells.

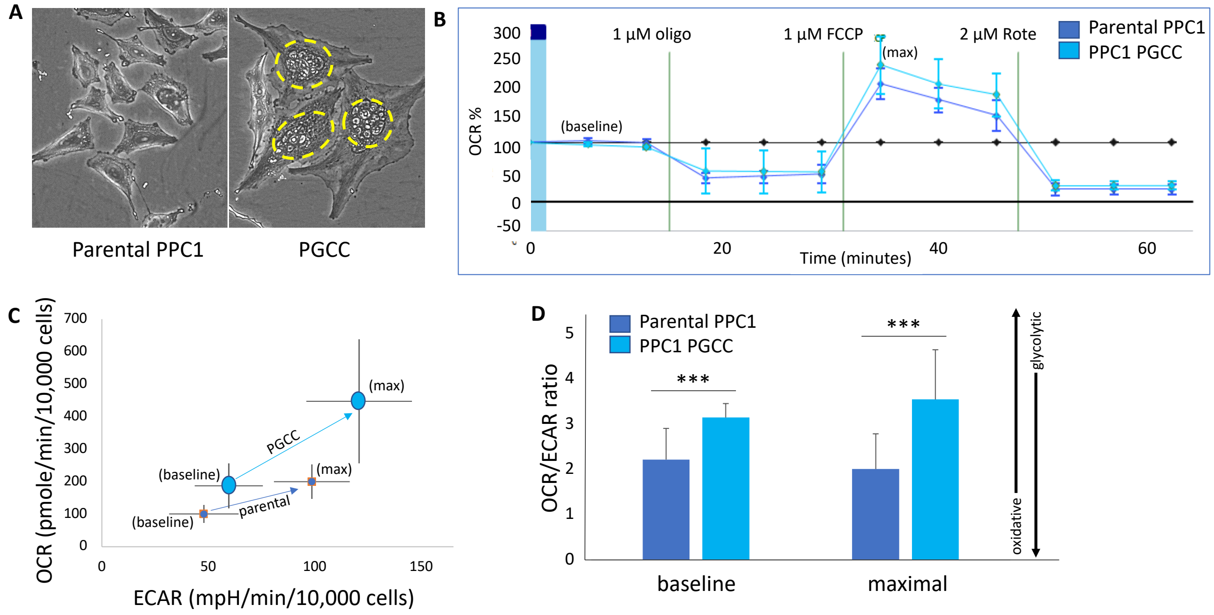

Supplementary Figure S1. Metabolic analysis of parental and PGCC from PPC1 cells. (A) Representative 20X image of parental PPC1 cells and PGCC with yellow outline denoting nuclear expansion in PGCC. (B) Seahorse metabolic traces show robust mitochondrial activity for PGCC. Both PGCC and parental cell traces were normalized to self-baseline and the resulting patterns of mitochondrial activity under Seahorse testing are similar. (B, C) PGCC have a more oxidative metabolism, with normalization to cell number enhancing the trend (B) which maintains significance even after OCR/ECAR ratio analysis (C). High extracellular acidification without high glucose consumption suggests a more glycolytic metabolism, which PGCC do not demonstrate at baseline or at maximal OCR compared to parental cells. ***$p<0.001$. *Supplementary methods:* For seahorse analysis, PPC1 cells were plated 8×10^5 in 100 mm plates and irradiated the following day. After two days PGCC were captured, counted, and plated at 10,000 per well into 46 wells of a 96 well plate. Parental PPC1s were trypsinized and counted to plate 10,000 per well into 46 of the remaining wells of the 96 well plate, leaving the four corner wells empty. The standard XF96 Metabolic Flux Analyzer (Agilent Seahorse Technologies, Santa Clara, CA) protocol was performed, injecting oligomycin, FCCP, and Rotenone at prescribed intervals and measuring oxygen consumption rate (OCR) and extracellular acidification (ECAR) over time. Wave V2.6 software was used to normalize each cell type to its own baseline in Supplementary Figure 2B. Excel software was used to analyze the baseline and maximal OCR and ECAR for each cell type, and the resulting ratio which broadly describes metabolic phenotype as more oxidative phosphorylation or glycolysis.

Supplementary Figure S2. Normalization of LC/MS data.

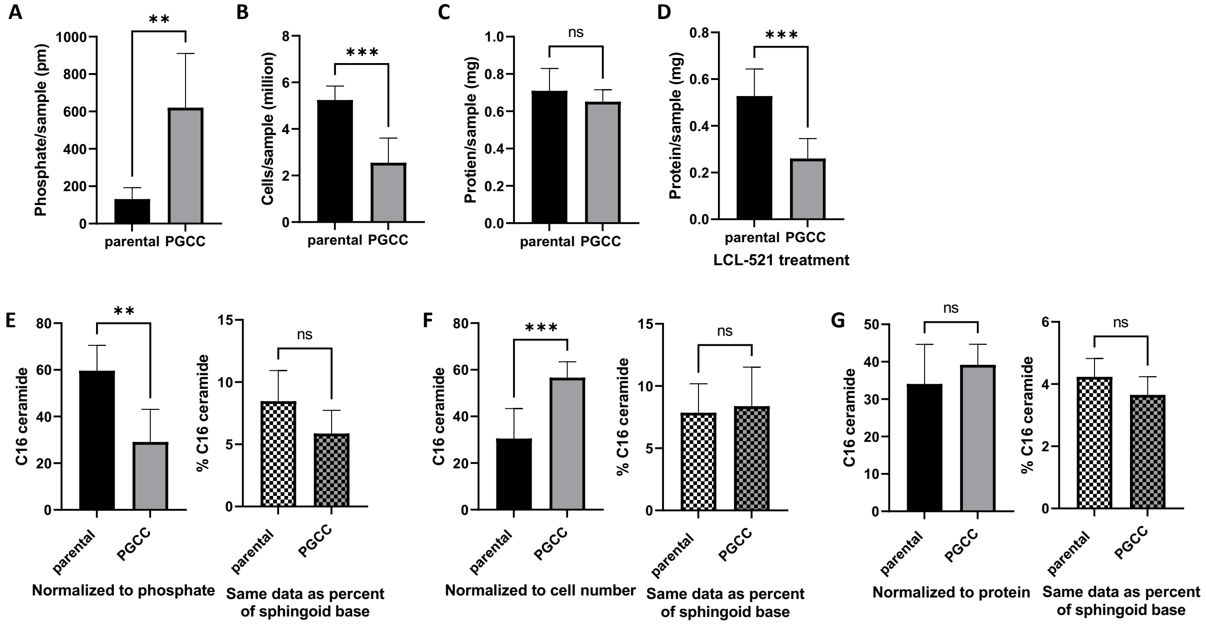

Supplementary Figure S2. Normalization of LC/MS data. Parental PPC1 and PGCC differ in amounts of phosphate (A) and cell number per sample (B). Protein was not significantly different between PPC1 and their PGCC (C) but protein content in PGCC decreased significantly upon ASAH1 inhibition (D). Differences of phosphates, cell number and protein in samples lead to inconsistent normalization (E-G left panels), requiring data to be normalized to percent of total sphingoid base per sample.

Supplementary Figure S3. LC/MS analysis of sphingomyelin and ceramide

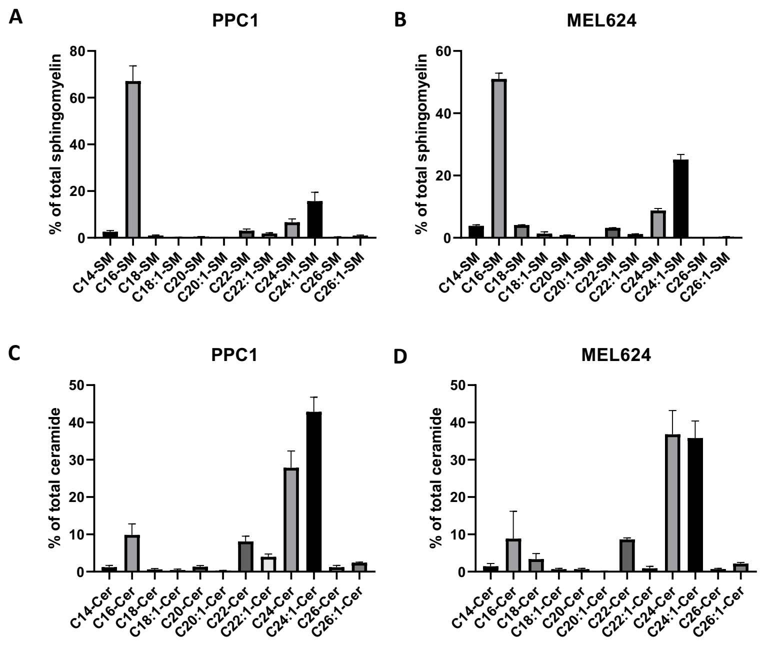

Supplementary Figure S3. LC/MS analysis of sphingomyelin and ceramide. PPC1 and MEL624 cells were analyzed for both sphingomyelin (A, B) and ceramide (C, D). Data represent the average +/- standard deviation from 3 independently performed experiments with triplicate samples.

Supplementary Figure S4. Distribution of ceramides in PPC1 cells with modulation of CerS6 and p53

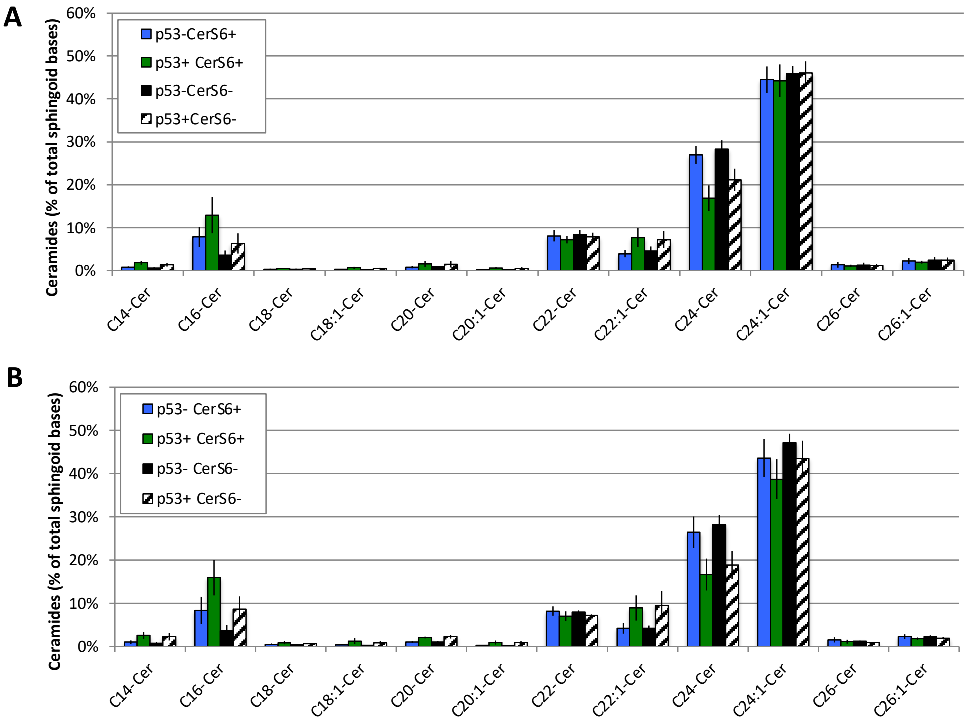

Supplementary Figure S4. Distribution of ceramides in PPC1 cells with modulation of CerS6 and p53. PPC1 cells (A) and PPC1-derived PGCC (B) were analyzed by LC/MS analysis. Knockdown of CerS6 results in significant decreases in C_{14}- and C_{16}-ceramide ($p<0.0001$). Expression of p53 significantly increases C_{14}-, C_{16}- and $C_{22:1}$-ceramide with a concomitant decrease in C_{24}-ceramide irrespective of CerS6 expression status ($p<0.0001$). Data are calculated as described in the Materials and Methods and represent the average +/- standard deviation from 3 independently performed experiments with triplicate samples.

Supplementary Figure S5. Raw Western blot data

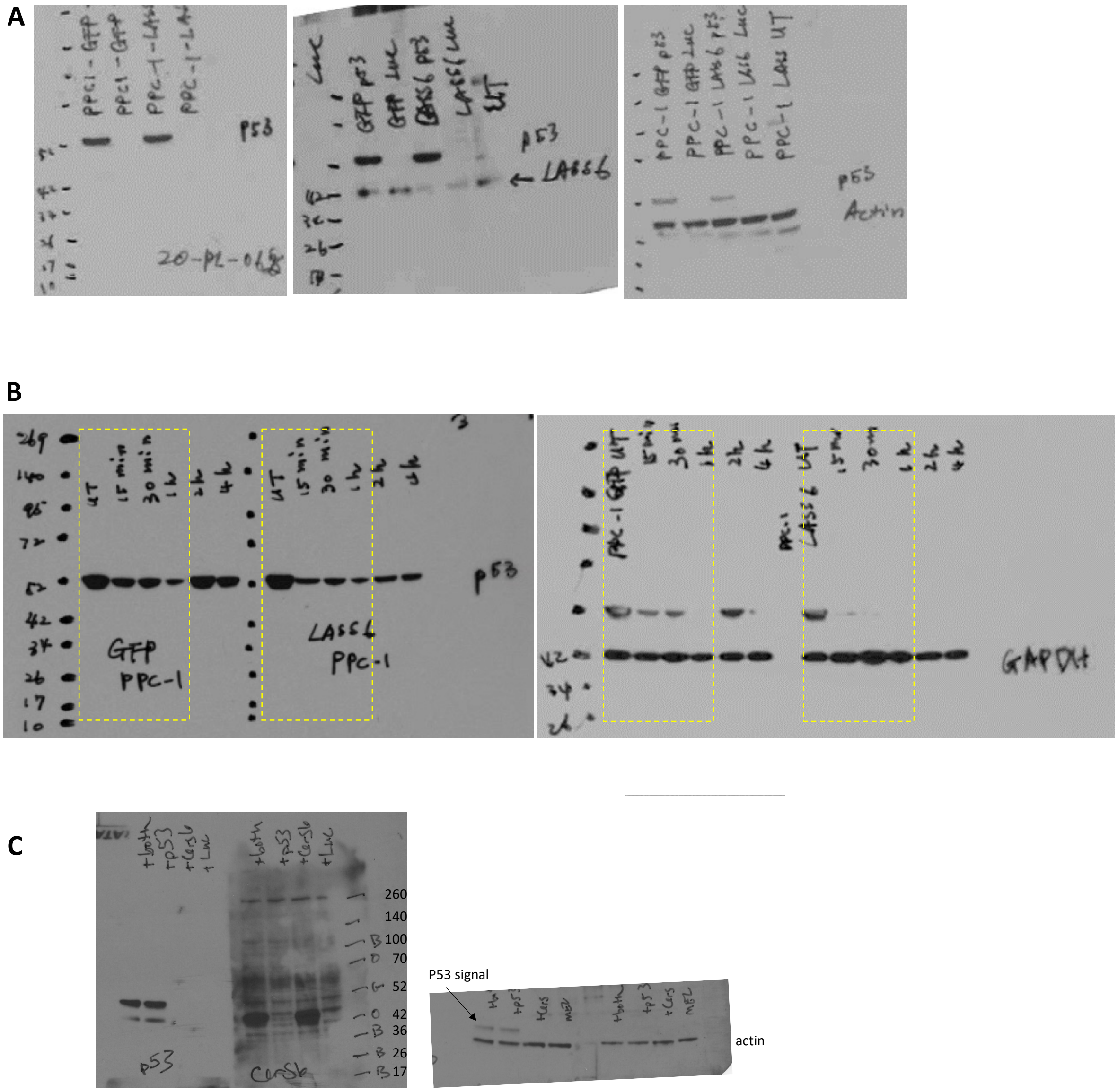

Supplementary Figure 5. Raw Western blot data with molecular weight markers. (A) Full blots for Figure 3A. (B) Full blots for Figure 3C. Portions shown in the figure are outlined in yellow.(C) Blots for Figure 5A.

Supplementary Table S1. LC/MS analysis of ceramide in parental PPC1 cells

	parental mean (st.dev)	parental+LCL521 mean (st. dev)	p value
C14-Cer	1.12 (.017)	0.79 (0.10)	***
C16-Cer	4.23 (0.59)	3.75 (1.07)	ns
C18-Cer	1.18 (0.44)	1.32 (0.46)	ns
C18:1-Cer	1.17 (.064)	0.80 (0.57)	ns
C20-Cer	0.65 (0.16)	0.97 (1.20)	**
C20:1-Cer	0.12 (0.04)	0.14 (0.04)	ns
C20:4-Cer	0.07 (0.11)	0.01 (0.01)	ns
C22-Cer	5.61 (1.04)	8.84 (2.42)	**
C22:1-Cer	1.70 (0.26)	1.62 (0.50)	ns
C24-Cer	41.2 (5.94)	38.0 (7.48)	ns
C24:1-Cer	25.9 (2.59)	29.6 (3.29)	*
C26-Cer	3.38 (2.73)	3.91 (1.47)	ns
C26:1-Cer	4.31 (1.12)	8.53 (0.81)	****
total ceramide	90.6 (2.28)	98.3 (0.88)	****
Sphingosine	9.21 (2.28)	1.62 (0.90)	****
S1P	0.15 (0.11)	0.03 (0.02)	**

Supplementary Table 1. Distribution of sphingoid bases within the ceramide/sphingosine/S1P circuit in parental PPC1 cells at baseline and after 5 hours of LCL521 treatment. *p<0.05. **p<0.01, ***p<0.001, ****p<0.0001. Data are calculated as described in the Materials and Methods and represent the average +/- standard deviation from 3 independently performed experiments with triplicate samples.

Supplementary Table S2. LC/MS analysis of ceramide in PPC1-PGCC

	PGCC mean (st. dev)	PGCC+LCL521 mean (st. dev)	p value
C14-Cer	1.04 (0.08)	1.34 (0.07)	****
C16-Cer	3.65 (0.59)	5.32 (0.99)	***
C18-Cer	1.72 (0.32)	3.18 (0.74)	****
C18:1-Cer	0.90 (0.11)	1.18 (0.23)	**
C20-Cer	0.97 (0.15)	2.01 (0.36)	****
C20:1-Cer	0.18 (0.03)	0.37 (0.10)	****
C20:4-Cer	0.01 (0.01)	0.01 (0.01)	ns
C22-Cer	7.16 (0.81)	8.99 (0.67)	****
C22:1-Cer	1.31 (0.17)	2.06 (0.44)	***
C24-Cer	49.7 (2.57)	36.1 (6.29)	****
C24:1-Cer	20.6 (1.42)	29.7 (3.75)	****
C26-Cer	4.01 (0.51)	2.81 (0.51)	***
C26:1-Cer	5.77 (0.82)	6.43 (0.47)	ns
total ceramide	97.0 (0.93)	99.4 (0.36)	****
Sphingosine	2.82 (0.93)	0.60 (0.23)	****
S1P	0.14 (0.04)	0.06 (0.01)	****

Supplementary Table 2. Distribution of sphingoid bases within the ceramide/sphingosine/S1P circuit in PPC1 PGCC cells at baseline and after 5 hours of LCL521 treatment. *$p<0.05$. **$p<0.01$, ***$p<0.001$, ****$p<0.0001$ "Data are calculated as described in the Materials and Methods and represent the average +/- standard deviation from 3 independently performed experiments with triplicate samples".
